# Opportunities and Challenges for an Organizational Digital Public Health Strategy in a Provincial Public Health Program in Canada: Qualitative Description of Practitioner Perspectives

**DOI:** 10.2196/72588

**Published:** 2025-08-12

**Authors:** Ihoghosa Iyamu, Devon Haag, Anna Carson, Ivy Wang, Colin King, Ian Roe, Kristy Kerr, Sofia Bartlett, Geoffrey McKee, Mark Gilbert

**Affiliations:** 1 BC Centre for Disease Control, Vancouver, BC, Canada; 2 School of Population and Public Health, University of British Columbia, 2206 East Mall, Vancouver, BC, V6T 1Z3, Canada, 1 604 707 5619; 3 Independent Consultancy, Vancouver, BC, Canada; 4 The ClearView Group - Solutions for Health, Delta, BC, Canada; 5 Pacific Public Health Foundation, Vancouver, BC, Canada

**Keywords:** digital health, digital public health, implementation science, digital health strategy

## Abstract

**Background:**

The digital transformation of health services accelerated during the pandemic. While “digital health” strategies were created, they paid minimal attention to public health services like health promotion, disease surveillance, emergency preparedness, and health protection.

**Objective:**

This study aimed to inform a digital public health (DPH) strategy at the British Columbia Centre for Disease Control (BCCDC) and explored public health practitioners’ perspectives on challenges and opportunities of integrating digital technologies into public health functions within the organization.

**Methods:**

In this qualitative description, we conducted 18 focus groups (FGs) between January and June 2023, drawing practitioners from 9 organizational subunits of the BCCDC including population and public health, environmental health, clinical services, vaccine-preventable diseases, communications, knowledge translation, data analytics, and Indigenous health (2 FGs per subunit). Discussions explored practitioners’ application of digital technologies in their public health work, focusing on challenges encountered during implementation (current state FGs) and perceived opportunities (future state FGs). Sessions were audio-recorded, and detailed field notes were taken. Thematic analysis was conducted, comparing perspectives across groups using constant comparative techniques.

**Results:**

We identified 3 themes. First, “bridging existing inequities—an opportunity and a challenge contingent on public trust” described participants’ excitement about opportunities for DPH to disrupt historical inequities if centered on trust and reconciliation, while recognizing current digital transformation efforts risk exacerbating existing inequities with the digital divide. Second, “a sense of disconnect between “digital” and “public health” functions” described perceptions of DPH as being out of scope of core public health duties, requiring new competencies and navigation of complex organizational policies for which support is suboptimal. Third, “balancing the need for responsive DPH with necessary reactivity” highlighted practitioners’ yearnings for a proactive DPH strategy rather than current issue-based reactive approaches. Participants suggest that a centralized systematic program can help achieve this goal.

**Conclusions:**

A cohesive, systematic, and proactive organizational strategy for DPH is critical to enable equity-focused digital transformation. Such a strategy can bridge perceived disconnects between digital and public health functions through organizational supports like competency development and streamlined policies that can better support public health practitioners to integrate digital technologies into their work.

## Introduction

Uptake of digital technologies for public health functions accelerated in the past decade, especially during the COVID-19 pandemic [1-3]. These technologies have been used to streamline real-time data processing, analysis, and reporting for health surveillance and emergency response [1,2,4]. They have also been used to expand the reach of contextually relevant and personalized health promotion and protection services through social media, mobile apps, and other similar technologies [1,5-7]. In 2022, Canada’s Chief Public Health Officer identified medical and digital health technology as a key part of strengthening Canada’s public health systems, recognizing its potential to improve the speed, efficiency, and impact of public health programs [8]. The strategy includes using digital technologies to strengthen early warning systems, improve supply chains and laboratory services through real-time tracking, support evidence-based decision making, and promote adherence to public health interventions via social media [8,9]. At the same time, it highlights the importance of carefully evaluating these technologies in light of legal and ethical concerns [8].

Current digital health strategies primarily focus on clinical applications, often overlooking the broader public health implications of digital transformation [10]. For instance, British Columbia’s digital health strategy emphasizes a trusted, digitally enabled health system aimed at patient empowerment, provider experience, system connectivity, and operational efficiency—largely within clinical settings [10,11]. However, the rapid adoption of digital technologies across sectors like commerce, education, and social services has significant public health implications [10,12]. Social media and the digital information environment also contribute to health inequities, particularly through misinformation and disinformation, which disproportionately affect the youngest, oldest, and least digitally literate populations [10,12-14]. Digital access and literacy influence key health determinants, shaping employment, education, and income, which in turn affect health behaviors, access, and outcomes [10,12,15,16]. A recent expert consensus highlights how digital transformation intersects with social, commercial, and political determinants of health [17]. Recognizing this, BC’s 2024 Population and Public Health Framework formally identifies the digital environment as a key determinant of health requiring public health action [18].

Yet, few public health agencies have developed digital public health (DPH) strategies that not only consider the role digital technologies play in strengthening public health functions, but also better position them to respond to the public health implications of society’s digital transformation as described [10]. This project was conducted as part of efforts to inform a DPH strategy within a provincial public health organization in Canada. This provincial organization is responsible for providing health surveillance, disease and injury prevention, diagnosis and treatment of diseases of public health importance, health promotion (including healthy public policy), and emergency response functions. This project was conducted in part to align organizational public health action with the broader British Columbia digital health strategy and the 2024 British Columbia population and public health framework, seeking to better position the organization and its workforce to address this evolving public health need. Therefore, we explored public health practitioners’ perspectives on the challenges and opportunities of applying digital technologies to public health functions within our organizational context.

## Methods

### Study Design

This was a qualitative description of public health practitioners’ perspectives on the opportunities and challenges of leveraging digital technologies to operationalize public health functions [19]. Our aim was to generate meaningful insights to inform the deliberations regarding the development of a DPH strategy within our organization. Similar qualitative approaches have been used to guide health services planning and policy formulation [20]. Our inquiry was grounded in a pragmatic and constructivist orientation, enabling us to draw from relevant theory while remaining participant-driven [21]. We built on the British Columbia Centre for Disease Control’s (BCCDC) strategic plan, which identifies “promoting digital health and clinical services” as a transformational priority, situating our study within the context of organizational change [10,22]. To inform our exploration of contextual factors shaping digital transformation, we drew on Weiner’s theory of organizational readiness for change, with attention to organizational policies, strategic priorities, perceived value of the change (change valence), and available resources [23]. Our working definitions of DPH were derived from a scoping review by our research team, alongside an internal review and national consultation process -Textbox 1 [22].

The project was led by public health practitioners (DH, GM, IR, II, and MG) with experience designing, implementing, and evaluating DPH interventions within the organization in collaboration with relevant health systems partners [24]. The research team also included private sector consultants with expertise in digital transformation across various health contexts (IW and CK) and public health researchers with qualitative research experience in applied health systems settings (II and AC). All authors were part of a DPH working group established with representation across the organization to guide the implementation of the project.

We acknowledge that our diverse professional roles and previous engagement with digital transformation influenced our philosophical stance and study design. Our constructivist approach was informed by an understanding that participants’ perspectives are shaped by their specific roles, work environments, and organizational objectives [19]. To mitigate potential bias and enhance reflexivity, data collection was conducted by consultants external to the organization, and analysis was led by an independent qualitative researcher who maintained a hands-off approach to support analytic independence. The full team engaged in regular reflective discussions on the emerging findings, using these sessions to critically examine how our positionality as insiders and researchers may have influenced interpretation. These ongoing reflective practices were integral to maintaining transparency and rigor in our approach.

Textbox 1. **Operational definition of digital public health.**

Definition of digital public health

- Digital public health is the use of digital technology to transform the delivery of public health functions in people-centered ways that optimize health outcomes for all. It brings together expertise in health, technology, and other disciplines to improve the health of populations, while addressing modern public health challenges that are amplified through the rapid and widespread uptake of digital technologies.
- Digital public health uses digital technologies to design, implement, evaluate, and scale up interventions to address public health challenges and promote health equity. It includes a wide range of digital technologies and applications, including data analytics, mobile health, social media, virtual health, and others.

### Study Setting, Sampling, and Recruitment

This project was conducted at the BCCDC, a program of the Provincial Health Services Authority responsible for provincial and national leadership in disease surveillance, detection, treatment, prevention, and health promotion. The program is organized into subunits called service areas including clinical prevention services and its 2 clinical programs (sexually transmitted infections and tuberculosis clinics); data and analytics services (DAS); environmental health services (EHS); immunization program and vaccine-preventable diseases; public health response (PHR); Population and Public Health and cross-cutting programs including the Chee Mamuk Indigenous Health Program and the communications and knowledge translation (KT) Program. This project was informed by broader organizational strategic planning goals of promoting digital health and clinical services.

Participants were recruited from across key organizational subunits (service areas and programs) to ensure a range of operational perspectives on DPH. Recruitment followed a purposive sampling approach, with invitations shared through team leads and internal email lists. All staff within the selected units were eligible to participate, and participation was voluntary. Due to privacy and organizational sensitivities, particularly given the small size and specialized nature of many teams, we do not report detailed participant characteristics such as roles or years of experience.

### Data Collection

Between February and April 2023, we conducted 2 rounds of focus groups (FGs-18 in all) with each of the service areas as described. FGs lasted an average of 52 minutes (range: 39‐64 min). In the first round, we explored challenges with applying digital technologies to public health functions in the current organizational context. In the second round, we explored potential opportunities and priorities for a desired future state of DPH within the organization. FG guides were adapted to each service area, with initial sessions informing slight adaptations to ensure that discussions elicited context-specific insights while maintaining comparability across groups Multimedia Appendix 1. FGs were facilitated by (DH, IW, and CK) who have experience leading group discussions. FGs were conducted on Microsoft Teams and all sessions were auto-transcribed, with transcripts reviewed for accuracy to mitigate potential technology-related issues. Detailed field notes were also recorded during and after each FG.

### Ethical Considerations

This project did not require formal ethics approval as it was classified as quality improvement given that it was under the jurisdiction of a public health authority and did not have research as a primary goal [25]. However, we followed basic ethical principles [26]. We obtained client consent, including verbal assent for recordings. To protect privacy, no identifiable information was collected. Participants could skip questions or withdraw at any time. No honorarium was provided, as FGs occurred during paid work hours.

### Data Analyses

We conducted a reflexive thematic analysis on the combined dataset from both rounds of FGs, following Braun and Clarke’s recommendations [27,28]. First, verbatim transcripts and field notes from both rounds of FGs were reviewed for accuracy and edited as necessary. All transcripts were imported into QSR NVivo (version 12) for data management and analysis [29]. The initial coding was performed by a primary analyst (AC), who closely read and reread transcripts to identify salient ideas and patterns in the data. A codebook was developed inductively and iteratively refined through regular consultations with the DPH working group Multimedia Appendix 2. A second analyst (II) reviewed the coding tree and assigned codes to ensure accuracy.

Throughout the coding and analysis process, we held weekly meetings with the DPH working group to discuss emerging codes, reflect on interpretive questions, and consider how organizational context influenced participants’ perspectives. We used triangulation across field notes, participant quotes, and group discussions to ensure credibility and conducted crosstabulations to explore patterns across different roles and areas of practice. Themes were developed, refined, named, and described through a process that balanced theoretical sensitivity with fidelity to participants’ voices. The final themes reflect the contextual realities, expectations, and concerns of practitioners engaged in DPH-related initiatives. In line with the qualitative descriptive approach, our analysis focused on capturing rich, context-specific insights without aiming for data saturation [19]. Our reporting adheres to the consolidated criteria for reporting qualitative research (COREQ) Checklist 1 [30].

## Results

### Description of FG Participants

Table 1 describes participants recruited from various service areas and programs in 2 rounds of FGs. Overall, there were 60 unique participants across 18 FGs, representing 6 service areas and 5 programs within BCCDC. Participants worked at varying levels including frontline clinical services, middle level, and executive-level management.

We identified 3 themes which are described in detail below, including representative quotes as appropriate.

**Table 1.** Description of focus group participation by Sub-Unit and round–British Columbia Centre for Disease Control (2023).

Focus group	Number of participants	
	Round 1	Round 2
Clinical prevention services line–sexually transmitted infections program	8	7
Clinical prevention services line-tuberculosis program	3	5
Data and analytics services line	6	5
Environmental health services	5	6
Immunization program and vaccine-preventable diseases	3	4
Public health response	6	4
Population and public health	4	10
Chee mamuk indigenous health program	9	4
Communications and knowledge translation programs	3	4

### Bridging Existing Inequities: An Opportunity and a Challenge Contingent on Public Trust

Practitioners described digital transformation as an opportunity to improve equity of public health services among historically marginalized populations. This was especially recognized for Indigenous peoples in Canada for whom additional opportunities for reconciliation exist. Practitioners emphasized that realizing the potential of digital transformation is contingent on establishing public trust which is influenced by the reach and effectiveness of current DPH efforts, the accessibility of digital services and resources, the timeliness of current efforts to bridge existing digital divides, and perceived benefits of digital transformation to various populations. Priorities for equity and reconciliation were especially emphasized for Indigenous communities who may be marginalized by current digital strategies. One participant said

*The truth of the matter is, there’s a lot of communities who are still existing on those older systems and like to me, it’s I guess for where I would like to currently sit on this conversation, is sitting in that tension of those who have and those who do not have, because at the end of the day, the people who are most going to pay for not being digitally connected are Indigenous people and that’s already knowing like we literally already know that and none are. But in the health systems, we’re actually going more to virtual and digital and we’re ensuring that we leave Indigenous people out*.

[Indigenous health program, Session 2]

While recognizing the potential for increased accessibility, reach, equity, effectiveness, and efficiency of public health services through digital transformation, service areas emphasized concerns about further marginalizing populations without dependable digital access and literacy. Practitioners described scenarios where Indigenous, rural, and remote communities, people with lower socioeconomic status, and people experiencing language barriers or visual impairments are already being marginalized by our current digital transformation efforts. For example, one participant described a situation where Indigenous Elders seeking vaccines were caught in a technological dilemma

*We had elders that were reaching out to the 1‐800 number looking for vaccination. Only to call the number, sit on the line for 45 minutes, and then for them to get on to the line of like saying, “Hey, you have to go to this website” and you’re like, “Well, we don’t have a laptop, nor do we have Internet. We were told to call this number.” And they’re like, “Yeah, you have to go to this website.” And so they were basically just pointing at each other.*

[Indigenous health program, Session 1]

Carefully considering the benefits of digital transformation of public health alongside limitations of the digital divide was described as an important way to avoid creating an inherently inequitable 2-tiered system. Practitioners also suggested that fostering trust in DPH is dependent on broader sociocultural contexts. They described various negative community perceptions of public activities including data collection through intervention, to planning implementation and KT. The need to address these perceptions in meaningful ways was described as fundamental to the organization’s ability to make progress within the fast-paced technological landscape

*Privacy is a constant struggle and it feels like there’s often the implicit assumption that we are trying to use the data to do terrible things and you know changing the conversation and it’s starting to change for sure, but changing the conversation to reframe it as the things we can do to benefit the population when the data exist and are accessible to us as opposed to the, you know, the various purposes that that we might put the data to you and you know if I had a blue sky world, you know that would be the first thing that everybody thought of when we asked for data or made a request for data was “wow, think of what good these people could do if they had access to those data.*

[EHS, Session 2]

Practitioners highlighted the importance of recognizing how socioeconomic status, geographical location, and access to health care services can both facilitate and hinder public trust in DPH, encouraging careful consideration of DPH’s role to address the potential harms of the broader digital transformation of society and new inequities resulting from the process (ie, the digital determinants of health)

*It would be a miss if we didn’t consider our mandate to address some of the harms and facilitators that there are from digital technology adoption and the broader community in the work that we do. I mean just looking at how certain technologies-through inequitable uptake- can exacerbate underlying inequities within society and how that addresses or how that can exacerbate the social determinants of health.*

[Population and public health, Session 2]

Practitioners also emphasized the need to provide concurrent non-digital options for public health interventions to promote equity. The following quote provides important context on addressing the digital divide as a 2-fold process

*The digital divide, I would characterize as two separate issues. The first one is people who just simply do not have digital access at all and that could be anything from having their own personal contact number, but it could also be like not having financial resources to have Internet connectivity. Because even people who have devices actually often do not have access because of the cost of data in Canada, or they don’t actually have consistent access to the Internet… And so, I think that this is actually something that’s a very kind of important gap and challenge for us to be thinking about, but also acknowledging. And then the second part of the digital divide is people who actually have access to these things, but do not have the digital literacy to be able to actually fully utilize them, and I would put a bunch of people into that kind of category. You know, myself included*.

[Sexually transmitted infections, Session 2]

Practitioners suggested that implementing strategies to address these issues can foster trust building among diverse populations, potentially enhancing the acceptability, efficacy, and efficiency of DPH.

### A Sense of Disconnect Between “Digital” and Public Health Functions

The second theme highlighted a perceived disconnect between the staff’s daily work and the envisioned or idealized practice of “doing DPH work.” Practitioners described how engaging with digital health and technologies was often viewed as “being in excess of assigned roles.” While often described with some frustration, practitioners also expressed excitement about the potential of DPH skill sets and practices to improve the reach, efficiency, and effectiveness of internal and external services of the organization. For example, this participant said

*I’m kind of thinking, wow, if we had a tool to do this, that would be great. I have all these potential future uses in mind now but I have no idea if there is such a thing. But you know, if there’s something that could help us do the work better or, you know, in new ways and reach more people, then that’s really exciting.*

[PHR, Session 1]

Practitioners described being unable to effectively formulate digital solutions to health systems problems they encounter. They emphasized needing in-service training and centralized supports to address competency gaps. These competency gaps were described as contributory to feelings of frustration with systems inefficiencies which exacerbate current workloads:

*My experience trying to submit my [cloud-based data platform] application was excruciating. Like I’m a pretty smart person, but not having expertise made that a horrible, very, very long process. Whereas if I had someone that was used to doing that, skilled in doing that, they could have sat down with me and spent an hour. So, trying to make each individual group become an expert, or at least skilled in doing this as opposed to having central resources that already have those skills and do it all the time, I think is really inefficient and it leaves opportunities on the table.*

[EHS, Session 1]

As described, practitioners acknowledged competency gaps but also emphasized the role of centralized support in filling capacity gaps for DPH services given current workloads and a sense that DPH services are not a core responsibility. For example, this participant said:

*We can give information, but it’s not on us because we literally don’t have the capacity, like we just don’t. And it would make sense […] like, where’s that digital team?*

[Indigenous health program, Session 2]

Practitioners also suggested creating novel positions with technology-specific job descriptions, with comparable remuneration rates to similar roles in the private sector. This was described as a prerequisite for effective DPH transformation, involving a re-evaluation of current human resource classification systems and securing additional funding

*I think that is one thing that basically requires creating a new stream of jobs within BCCDC […]. Just say like we don’t have these jobs and have been working on even bringing one person on board and we have been just kind of like trying to pigeonhole these people into our existing positions and that also kind of like goes back to our comp and class system, which is a little bit archaic. It takes quite a bit of time to get a new position classified and so on, so it’s at multiple levels. First is just kind of like the realization to think through, these are skills that are needed for the future of work that we do at [BCCDC]… and then the second is creating these job descriptions, having funding allocated to hire these people and then create an ecosystem–whether it’s through partnerships with academics and the scientists here to provide that environment where people are successful in implementing their skills and providing solutions to our problems.*

[DAS, Session 2]

Practitioners suggested internal capacity-building to effectively use existing digital technologies (eg, limited knowledge of Microsoft Office suite tools, graphic design, and tools to support building and maintaining internet-based courses and manuals) while prioritizing the need to hire in-house resources to support more advanced digital and analytics projects.

### Balancing the Need for Responsive DPH With Necessary Reactivity

This theme emphasizes the need to balance systems’ reactivity with responsivity through an approach that fosters a proactive DPH strategy across service areas and programs. Here, reactivity was used to describe issue-based and unplanned implementation of digital technology in response to emergent public health issues as compared with responsivity, which describes proactive and planned strategies for considering DPH within broader public health responses. Practitioners described creative, adaptive, and individual approaches to implementing digital tools and programs, both internally and externally, and described needing a centralized approach at the BCCDC to guide DPH transformation. Practitioners critiqued overly reactive systems built on assumptions of public health’s inherent function of reacting to emerging threats. This was said to result in multiple and inconsistent communication channels with public and providers, potentially limiting the organization’s public health impact. For example, one practitioner said

*So, in terms of messaging information and from a communicable disease, rapid response perspective, being able to push out consistent messaging on a new emerging [threat], whether it’s a measles outbreak or an upcoming heat [wave] because we don’t have that one-stop shop […] It’s just–there’s too many ways to get slightly different information and then we end up potentially not having the most impactful inputs or support to that group of providers.*

[PHR, Session 2]

This reactive approach often leads to rapidly developed DPH interventions described as frequently untested, duplicative of previous efforts, and contributing to fragmented DPH systems. These fragmented systems were described as potentially wasteful and challenging to integrate into existing systems, ultimately undermining confidence in DPH initiatives. To reduce this reactivity, one participant suggested promoting broader access to and understanding of emerging digital tools across the organization

*It’s not just someone to help create that vision with using all these tools, but also the access to the right resources in a timely manner to learn what else is coming. I could dream up a lot of things, but I can only go so far because I don’t know what else is in the [IT subunit] world. The funny thing is, like the texting solution that worked on the COVID side, I was the technology expert, way back in the day, we had created the concept before, and it just sat on the shelf.*

[EHS, Session 2]

While the organization may not anticipate all potential technological shifts, setting the groundwork for information management (eg, privacy guidelines and standards for novel datasets), communication, and centralized support will provide needed foundations for service areas to work from. Perspectives of unclear DPH foundations are compounded by the current ad-hoc approach to DPH relying on staff motivation, as illustrated in the following quote about the lack of institutional knowledge in creating online courses for health care providers

*I mean, going back to the conversation we’re having about the courses that are built on [software name]. Like we don’t necessarily have any training. People just have taken it upon themselves to figure it out.*

[STI, Session 1]

Practitioners’ narratives emphasized the need for a systematic and centralized program to lead the organization’s effort in a more responsive and proactive approach to DPH. This also involves enhancing data, clinical, and operational interoperability among health authorities, governmental levels, and other health systems partners. Participants highlighted the challenges they experienced with provincial interoperability

*These...solutions within [health authority (HA1)] and then later on within [HA2] and then for the regular communicable disease and other diseases. We have [software 1] which is being used by one or two health authorities. Then we have [software 2] within [HA3] and then we have [software 3] within [HA1] and then a different system in [HA4]. Like we don’t have a good mechanism to capture information and then a mechanism to develop some of these solutions on the fly, because in public health you need to have some nimbleness, like when COVID emerged.*

[DAS, Session 2]

Interoperability challenges were also said to result from complex and inconsistent data access protocols, agreements, and platforms that are cumbersome to navigate at an interinstitutional level, while acknowledging ongoing work to address these barriers.

*There just isn’t provincially consistent and reliable structures for sharing data across groups, and we get hung up in, you know, information–like establishing information sharing agreements can take years in some cases, and it really impedes our ability to do the work that we need to do. So that’s kind of a constant frustration.*

[EHS, Session 1]

Practitioners highlighted opportunities to leverage and repurpose existing technologies and platforms across service areas, automate tasks (eg, data entry and cleaning, communication with patients and providers), and improve communication, policies, and training opportunities as potential solutions aligned with public health needs. As noted by this participant, lengthy approval processes do not align with priorities for nimble responsiveness or necessary reactivity.

*The thought is that there needs to be a generally rapid response if something is out there, and currently, because of the communication approval process, there’s very little that’s rapid. So, I think there’s a lot of like bits feeding into, you know, even if we do decide that we need to change how we manage mis- or disinformation, we’re not set up for success in that at the moment.*

[KT, Session 2]

Clinical staff also noted technological gaps that could alleviate frustrations in patient-facing services if addressed. Comprehensively integrating more flexible and automated services was highlighted as a change that could improve system responsivity. For instance, while acknowledging external constraints (ie, interinstitutional interoperability), the clinical tuberculosis program offered suggestions to improve internal responsivity through specific channels

*There’s the appointment reminders piece, like the automated appointment reminders piece. There’s the fact that we still mail things out. So, there’s no direct fax capability like there is with basically any EMR that you buy off the shelf. We don’t have a texting platform. We don’t have a scheduler that actually allows us to provide and schedule the patients in the way in which we would like to schedule them–like we have an incredibly rigid scheduler.*

[Tuberculosis, Session 1]

Practitioners emphasized needing robust mechanisms for information, data exchange, and collaboration among partners within the DPH landscape. This includes advocacy for standardized protocols and frameworks that facilitate seamless data sharing and communication among health authorities and government entities at various levels. However, prioritizing efforts to enhance the accessibility, timeliness, and utility of DPH tools (including digital clinical tools) is easier to achieve internally and will build in the desired system responsivity.

Participants also highlighted concerns about the sustainability of DPH transformation processes, including the infrequency of comprehensive and full-cycle planning that considers evaluation and knowledge translation for DPH interventions. Insufficient evaluation was said to limit the organization’s ability to demonstrate impact and secure ongoing funding for DPH interventions. For example, participants emphasized the importance of rigorous evaluation of DPH interventions

*That evaluation component I think is really, really important and something our team really wants to try and get sorted. But again, capacity is a huge component of that for us, so.*

[KT, Session 2]

*Whatever we decide to move forward with, we want to have a fairly robust evaluation strategy built into that to make sure it’s gonna be effective and be of use for the target population*.

[Immunization program and vaccine-preventable diseases, Session 2]

Addressing these issues requires a shift toward a more structured and comprehensive DPH transformation approach within the organization, integrating systematic DPH planning, program design with clear goals, effective implementation, evaluation, and knowledge translation. Moreover, establishing rigorous evaluation frameworks is essential to generating evidence on the efficacy and effectiveness (including cost-effectiveness) of DPH interventions, potentially enhancing their long-term success and sustainability. Participants considered the importance of interdisciplinary academic and industry partnerships in addressing these ‘full cycle’ approaches to DPH interventions

*There were folks at the University of Waterloo, and their computer sciences and health management system. And they wanted to work with us. But we got busy in the pandemic. Similarly, here at UBC and SFU. So, there are so many things–like they could develop apps, they could come up with completely new ideas because we don’t usually work in that environment. And our sphere of thought processes revolves mainly around epidemiology and statistics. And we are not engaging in computer science. And some of the digital people who work on some of these solutions from their point of view and if you bring it that kind of like cross-disciplinary approach, we may be able to come up with the solutions which are a little bit better suited than what we have right now*.

[DAS, Session 2]

## Discussion

### Principal Findings

In this qualitative descriptive study of practitioners’ perspectives on the challenges and opportunities of DPH, we identified 3 interconnected themes. These align with broader digital health strategies and reflect constructs from organizational change theory, including change valence, change efficacy, organizational norms and procedures, and perceptions of contextual resources [23,31].

First, practitioners’ expressed narratives about bridging existing inequities - an opportunity and a challenge contingent on public trust. This reflected practitioners’ optimism about the potential for DPH to advance equity, effectiveness, and reconciliation while being concerned about its capacity to widen the digital divide. This emphasized building and maintaining public trust as a foundational element of DPH. Second, practitioners reported a sense of disconnect between “digital” and public health functions. DPH was often viewed as outside the traditional scope of public health work, reflecting concerns about limited digital competencies and fragmented systems. Practitioners described needing centralized supports, targeted in-service training, and restructured human resource systems aligned with emerging DPH needs. Third, practitioners’ discourse regarding balancing the need for responsive DPH with necessary reactivity described challenges with overly reactive and somewhat ad-hoc approaches to digital transformation across the organization. Digital initiatives were often issue-specific and fragmented. These accounts reflect a reactive organizational culture, underscoring needed shifts toward more proactive, integrated approaches. Practitioners emphasized needing improved internal and external coordination within the DPH ecosystems. Effective communication, interoperability, and rigorous evaluation were identified as essential components of a sustainable DPH system.

Our findings contribute to the limited but growing body of literature on organizational transformation in public health settings [22,32]. While digital health implementation is often studied in clinical contexts, few studies have examined how public health organizations navigate structural, cultural, and operational shifts in the context of digital transformation [10]. Echoing findings from clinical settings, practitioners in this study expressed optimism about the potential of digital tools to improve access, coordination, and outcomes [32]. However, they also raised concerns about the absence of coherent organizational strategies, limited digital infrastructure, and persistent gaps in workforce capacity [33-37]. Our findings reinforce the need for digital transformation efforts to be accompanied by robust organizational capacity-building strategies. Gaps in digital competencies, which range from basic digital literacy to specialized technical skills, undermine practitioners’ ability to engage with and sustain digital initiatives [33,37,38]. Our findings also align with studies demonstrating public trust and engagement as a core part of digital transformation strategies and can be facilitated through proactive communication and transparent partnerships [33,35,37,38].

This study also illustrates tensions public health practitioners must navigate while facilitating the digital transformation of public health within broader societal digital transformation. Practitioner perspectives reflect a pre-existing institutional focus on equity and reconciliation [18]. However, perceived tensions emphasize calls for a techno-realistic equity-focused approach to digital transformation [9,39]. Further, practitioners’ sense of disconnect between DPH and core public health functions is explained by expressed concerns about change efficacy for transformation [23]. These concerns exist at both individual and system levels, echoing similar concerns identified in our prior research [23,38,40]. Change efficacy concerns also demonstrate potential misalignments between the prevailing organizational culture and a view of DPH as integral to core public health functions [23,31,37].

This misalignment may be reinforced by current issue-focused and ad-hoc approaches to deploying digital tools to address specific short-term public health concerns [37,38]. Other studies suggest this approach, combined with organizational bureaucracies, reinforces the apparent fragmentation of digital systems and limits the potential for digital transformation of public health [31,37,41]. Within this context, incentives for privacy and infrastructure workarounds that are dependent on an enthusiastic few continue to complicate perceived fragmentation [22,37,41]. These findings demonstrate the role of proactive leadership capable of shaping organizational norms, culture, and processes to support long-term digital transformation, while also navigating short-term operational demands [23,31]. Such efforts must support practitioners to see DPH as integral to long-term visions, rather than peripheral to their mandate.

### Implications for Public Health Practice and Research

Our study has implications for organizational DPH strategies. Given existing recommendations for people-centered digital health strategies guided by integrated leadership, financial, organizational, human, and technological resources, it is reassuring that our findings are well aligned with British Columbia’s broader digital health strategy which centers trust and equity [11,42,43]. Building on this will require better governance and collaboration among partners and teams across the organization and served communities to not only build trusted integrated systems but also prevent duplication of efforts. These efforts must occur alongside ongoing advocacy for equitable digital access and literacy [44,45]. Moreover, our findings of a disconnect emphasize the need for an integrated review of public health training programs and their curricula to more appropriately reflect the role of digital transformation in contemporary public health, starting with basic general digital competencies with opportunities for specialization [46]. Our findings emphasize the need for a cohesive strategy to train public health practitioners across the organization on an ongoing basis to ensure they are up to date with modern digital technologies. Our findings also highlight the need for a revamp of current hiring practices with updated job descriptions (especially in a transdisciplinary context) and new public health roles for digital specialists (eg, highly skilled data scientists, computer scientists, and artificial intelligence and machine learning experts) with clearer career progression plans and competitive compensation packages [37,42].

Finding the need to balance responsivity with necessary reactivity highlights longstanding tensions in public health that significantly affected the public health response during the COVID-19 pandemic [8,41]. While the pan-Canadian and the British Columbia digital health strategies seek to implement people-centered interoperable systems, our study suggests adapting and extending this same vision across the organization to include all digital systems and supports [41,42]. Such an approach will require a centralized DPH program with digital specialists to support effective communication across the organization, maintain a repository of digital interventions and identify opportunities for shared costs, support ongoing practitioner training, support surveillance and attention to the growing risk of digital determinants of health, support rigorous evaluation of DPH interventions and ensure governance of these systems [37]. However, we must be careful not to allow these resources to constitute barriers to innovation while helping to transition the organization from reactive to more proactive and people-centered DPH systems [42]. Effective implementation will require not only technical resources but also strong organizational readiness, including alignment between leadership, workforce capacity, and operational structures that can sustain digital transformation over time [23].

Findings from this study should be considered based on its strengths and limitations. First, this study uniquely focuses on practitioner perspectives in real-world DPH contexts. Much of the literature has focused on mainly clinical and health systems. Our study explores the realities of public agencies that are reliant on internal and external process and data flows necessary for public health action across varied jurisdictions. It also accounts for the growing role of digital determinants of health. Our study comprehensively explores experiences of practitioners within a provincial public health organization, reflecting perspectives from similarly sized organizations which support comparable mandates. However, care must be taken to transfer findings from this study to other jurisdictions, given the uniqueness of the organization (ie, a provincial role) and the health systems within which the organization is located. We have also not accounted for community and partner perspectives in this study and our recommendations. Further research is needed to appropriately capture these perspectives to inform a comprehensive DPH strategy. Research is also required to understand the models through which recommendations for centralized supports, ongoing training, and systems revisions will become operable.

### Conclusions

While practitioners share excitement about the opportunities for DPH to facilitate equitable, efficient, and reconciliatory public health action, there are shared concerns about DPH being out of scope of core public health work, digital competency gaps, and a reactive approach to digital transformation within the organization that results in fragmented systems that limit DPH’s potential impacts. Organizational DPH strategies are needed to support practitioners to achieve equity through DPH by providing a cohesive, systematic, and proactive approach to digital transformation through clear and careful communication with partners and communities, updated human resource and in-service training policies that support the organization with engaging and retaining the right digital talent, and creating centralized supports to ensure a more responsive approach to DPH.

## Supplementary material

10.2196/72588 Multimedia Appendix 1 Focus group discussion guide.

10.2196/72588 Multimedia Appendix 2 Codebook.

10.2196/72588 Checklist 1 COREQ (COnsolidated criteria for REporting Qualitative research) Checklist.

## Abbreviations

BCCDC: BC Centre for Disease Control
COREQ: Consolidated criteria for reporting qualitative research
DAS: data and analytics services
DPH: digital public health
EHS: environmental health services
FGs: focus groups
KT: knowledge translation
PHR: public health response
