# Supplementary material for: Opportunities and Challenges for an Organizational Digital Public Health Strategy in a Provincial Public Health Program in Canada: Qualitative Description of Practitioner Perspectives
Source: JMIR Public Health Surveill. 2025 Aug 12;11:e72588. doi: 10.2196/72588 (PMC12342690; doi:10.2196/72588)
Supplement: Multimedia Appendix 1 [file publichealth-v11-e72588-s001.docx]

# **Appendices**

**Appendix 1 – Focus group discussion guides**

**Focus Groups Session 1**

**Current State**

1. How are digital technologies being used in your program area?
2. Are there other digital health-based projects planned or underway? If so, please provide more information on:
   1. What it is
   2. Target audiences / who’s impacted
   3. Funding
   4. How has it been evaluated?

**Current Gaps or Challenges**

1. Are there any gaps or issues in how digital technologies are being used today in your program?
2. Are there any partnerships with other sectors that you’d like to develop to improve or expand the use of digital technologies in your program?
3. What other education or skills related to digital health do you feel would help to further utilize digital technologies in your program?
4. Have you experienced any challenges or barriers to integrating digital health technology in your program? (e.g. barriers related to digital health technology implementation)

**Focus Groups Session 2**

**Future State Opportunities**

Prompts for envisioning future DPH opportunities:

1. Opportunities to use digital technologies to better prepare, plan and respond to significant public health events / emergencies?
2. Rethink how Communicable Disease (CD) Control Manual is delivered?
3. Opportunities for digital technology to fill gaps highlighted by the COVID-19 pandemic?
4. What is needed to make better use of data to engage the public?
5. How else can digital technologies be used in response to the toxic drug crisis?
6. How else can digital health improve your work?

Note: these opportunities should be tool/technology agnostic. Think about functions and capabilities, rather than a specific tool/software.

**Appendix 2 – Codebook**

| Name |
| --- |
| BCCDC Specific |
| Current Interventions |
| Future Interventions |
| Priorities |
| High Priority |
| Low Priority |
| Medium Priority |
| Designated or Intended Audience or Partners |
| External |
| Academic |
| Government |
| Patients and General Public |
| Rural and remote |
| Urban |
| Practitioners and Clinicians |
| Private and not-for-profit organizations |
| Internal |
| Features of DPH Interventions |
| Accessibility |
| Cost and Funding |
| Engagement and Collaboration |
| Equity |
| Digital Divide |
| Digital Literacy |
| Evaluation |
| Interoperability |
| Regulation and Privacy |
| Source |
| Indigenous Public Health |
| KT and Knowledge Mobilization |
| Misinformation and Trust |
| Public Health Area |
| Disease and injury prevention |
| Health Promotion |
| Policy Development |
| Surveillance and Detection |
| Treatment |
| Social media |
| Summarized Issues and Gaps |
| Summarized Partnerships and Opportunities |
| Technology |
| Data |
| Data Access and Linkage |
| Systems |
| Tools |
| Automation |
| BCCDC Website |
| Workforce and Human Resources |
| Education and training |
| Hiring and Market Forces |

**Appendix 3 – COREQ (COnsolidated criteria for REporting Qualitative research) Checklist**

Developed from:

Tong A, Sainsbury P, Craig J. Consolidated criteria for reporting qualitative research (COREQ): a 32-item checklist for interviews and focus groups. *International Journal for Quality in Health Care*. 2007. Volume 19, Number 6: pp. 349 – 357

| **No. Item** | **Guide questions/description** | **Reported on Page #** |
| --- | --- | --- |
| **Domain 1: Research team and reﬂexivity** |  |  |
| *Personal Characteristics* |  |  |
| 1. Interviewer/facilitator | Which author/s conducted the interview or focus group? | 8 |
| 2. Credentials | What were the researcher’s credentials? E.g., PhD, MD | 8 |
| 3. Occupation | What was their occupation at the time of the study? | 8 |
| 4. Gender | Was the researcher male or female? | 8 |
| 5. Experience and training | What experience or training did the researcher have? | 8 |
| *Relationship with participants* |  |  |
| 6. Relationship established | Was a relationship established prior to study commencement? | 8 |
| 7. Participant knowledge of the interviewer | What did the participants know about the researcher? e.g., personal goals, reasons for doing the research | Appendix 2 |
| 8. Interviewer characteristics | What characteristics were reported about the inter viewer/facilitator? e.g., Bias, assumptions, reasons and interests in the research topic | 6-7 |

| **Domain 2: study design** |  |  |
| --- | --- | --- |
| *Theoretical framework* |  |  |
| 9. Methodological orientation and Theory | What methodological orientation was stated to underpin the study? e.g. grounded theory, discourse analysis, ethnography, phenomenology, content analysis | 6 |
| *Participant selection* |  |  |
| 10. Sampling | How were participants selected? e.g. purposive, convenience, consecutive, snowball | 6-7 |
| 11. Method of approach | How were participants approached? e.g. face-to-face, telephone, mail, email | 6-7 |
| 12. Sample size | How many participants were in the study? | 8-9 |
| 13. Non-participation | How many people refused to participate or dropped out? Reasons? | NA |
| *Setting* |  |  |
| 14. Setting of data collection | Where was the data collected? e.g. home, clinic, workplace | 8 |
| 15. Presence of non-participants | Was anyone else present besides the participants and researchers? | 8 |
| 16. Description of sample | What are the important characteristics of the sample? e.g. demographic data, date | 10 |
| *Data collection* |  |  |
| 17. Interview guide | Were questions, prompts, guides provided by the authors? Was it pilot tested? | 8 (Appendix 2) |
| 18. Repeat interviews | Were repeat inter views carried out? If yes, how many? | NA |
| 19. Audio/visual recording | Did the research use audio or visual recording to collect the data? | 8 |
| 20. Field notes | Were ﬁeld notes made during and/or after the interview or focus group? | 8 |
| 21. Duration | What was the duration of the inter views or focus group? | 8 |
| 22. Data saturation | Was data saturation discussed? | NA (data saturation not required in interpretive description) |
| 23. Transcripts returned | Were transcripts returned to participants for comment and/or correction? | NA |
| **Domain 3: analysis and ﬁndings** |  |  |
| *Data analysis* |  |  |
| 24. Number of data coders | How many data coders coded the data? | 8-9 |
| 25. Description of the coding tree | Did authors provide a description of the coding tree? | Appendix 3 |
| 26. Derivation of themes | Were themes identiﬁed in advance or derived from the data? | 8-9 |
| 27. Software | What software, if applicable, was used to manage the data? | 8 |
| 28. Participant checking | Did participants provide feedback on the ﬁndings? | NA (advisory group used instead) |
| *Reporting* |  |  |
| 29. Quotations presented | Were participant quotations presented to illustrate the themes/ﬁndings? Was each quotation identiﬁed? e.g., participant number | 11-15 |
| 30. Data and ﬁndings consistent | Was there consistency between the data presented and the ﬁndings? | 11-15 |
| 31. Clarity of major themes | Were major themes clearly presented in the ﬁndings? | 11-15 |
| 32. Clarity of minor themes | Is there a description of diverse cases or discussion of minor themes? | 11-15 |
