# Supplementary material for: Opportunities and Challenges for an Organizational Digital Public Health Strategy in a Provincial Public Health Program in Canada: Qualitative Description of Practitioner Perspectives
Source: JMIR Public Health Surveill. 2025 Aug 12;11:e72588. doi: 10.2196/72588 (PMC12342690; doi:10.2196/72588)
Supplement: Multimedia Appendix 2 [file publichealth-v11-e72588-s002.docx]

**Appendix 2 – Codebook**

| Name |  |
| --- | --- |
| BCCDC Specific | Refers to conversations about DPH that are directly related to the BC Centre for Disease Control or its internal context. |
| Current Interventions | Existing DPH initiatives, technologies, or strategies already in use. |
| Future Interventions | Planned or proposed DPH interventions yet to be implemented. |
| Priorities | Topics or actions identified as needing attention or investment, and participants’ prioritization of these actions. |
| High Priority | Areas that are urgent or of top strategic importance. |
| Low Priority | Areas considered less critical or urgent. |
| Medium Priority | Areas with moderate importance or urgency. |
| Designated or Intended Audience or Partners | Groups or sectors identified as partners or target audiences for DPH efforts. |
| External | Entities or partners outside the organization. |
| Academic | Universities, research institutions, and scholars. |
| Government | Provincial, federal, or regional government bodies or policy makers. |
| Patients and General Public | Public populations targeted by DPH interventions. |
| Rural and remote | Populations or regions with limited access to centralized health services. |
| Urban | Populations or contexts within cities or urban health environments. |
| Practitioners and Clinicians | Public health professionals and healthcare providers. |
| Private and not-for-profit organizations | Businesses, tech companies, NGOs, or charitable organizations. |
| Internal | Within the organization (e.g., BCCDC teams or departments). |
| Features of DPH Interventions | Key characteristics or considerations associated with digital health strategies. |
| Accessibility | The ease of access to DPH tools, platforms, or services for different populations. |
| Cost and Funding | Financial considerations including budgets, affordability, and sustainability. |
| Engagement and Collaboration | Involvement of partners and cross-sectoral partnerships. |
| Equity | Conversations about the potential of DPH to bridge unfair health outcomes or exacerbate them. Includes conversations about unfair access and benefits of DPH across different groups and populations. |
| Digital Divide | Inequities in access to digital technologies and the internet. |
| Digital Literacy | Skills and knowledge required to navigate and use digital tools effectively. |
| Evaluation | Processes for assessing effectiveness, impact, and value of DPH interventions. |
| Interoperability | Ability of digital systems and tools to exchange and use shared information. |
| Regulation and Privacy | Legal and ethical aspects, including data protection and confidentiality. |
| Source | Where the information or intervention originated (e.g., report, meeting, literature). |
| Indigenous Public Health | DPH efforts that relate specifically to Indigenous populations and knowledge systems. |
| KT and Knowledge Mobilization | Activities that promote the use of evidence in public health practice. |
| Misinformation and Trust | Concerns about false information and public confidence in health systems. |
| Public Health Area | Core areas of public health where digital interventions are applied. |
| Disease and injury prevention | Preventing communicable and non-communicable diseases and injuries. |
| Health Promotion | Strategies that support healthy behaviors and environments. |
| Policy Development | Processes and outputs that shape rules, laws, or guidance. |
| Surveillance and Detection | Monitoring and early detection of health events or risks. |
| Treatment | Use of digital tools to support clinical care or therapy delivery. |
| Social media | Digital platforms used for communication, engagement, and outreach. |
| Summarized Issues and Gaps | Consolidated challenges or deficiencies identified in the DPH landscape. |
| Summarized Partnerships and Opportunities | Consolidated information about existing or potential collaborations. |
| Technology | Hardware or software used in DPH. |
| Data | Information generated, used, or managed in DPH. |
| Data Access and Linkage | Ability to retrieve and connect data across systems or sectors. |
| Systems | Infrastructure or networks that support DPH activities. |
| Tools | Specific applications, platforms, or products used in DPH. |
| Automation | Use of technology to perform tasks with minimal human input. |
| BCCDC Website | Conversations about updating or using the BCCDC's official site as a health promotion tool. Also includes conversations about challenges with the website. |
| Workforce and Human Resources | Public health personnel involved in or impacted by DPH. |
| Education and training | Discussions about educational and/capacity requirements for the workforce to effectively develop DPH. |
| Hiring and Market Forces | Recruitment and labor market dynamics affecting digital public health roles. |
